# An improved YOLOv5n algorithm for detecting surface defects in industrial components

**DOI:** 10.1038/s41598-025-94109-8

**Published:** 2025-03-21

**Authors:** Jia-Hui Tian, Xue-Feng Feng, Feng Li, Qing-Long Xian, Zhen-Hong Jia, Jie-Liang Liu

**Affiliations:** 1 College of Computer Science and Technology, Xinjiang University, Urumqi, 830046 China; 2 Xinjiang University Signal Detection and Processing Autonomous Region Key Laboratory, Urumqi, 830046 China; 3 Xinjiang Uygur Autonomous Region Research Institute of Measurement and Testing, Urumqi, 830000 China

**Keywords:** Defect detection, Attention mechanism, Dynamic snake Convolution, SIoU loss function, Engineering, Mathematics and computing

## Abstract

Due to the small defect areas and indistinct features on industrial components, detecting surface defects with high accuracy remains challenging, often leading to false detections. To address these issues, this paper proposes an improved YOLOv5n algorithm for industrial surface defect detection. The main improvements are as follows: the DSConv-CA module in the backbone network enhances the feature extraction capability, the Gold-YOLO structure replaces the original PANet structure in the neck to improve information fusion, and the SIoU loss function is adopted to replace the regression loss, further improving detection accuracy. Experimental results demonstrate that the improved YOLOv5n algorithm achieves a mean average precision of 75.3% on the NEU-DET dataset, which is 4.3% higher than the original model.

## Introduction

In the industrial production process, surface defect detection of industrial components is a key factor in improving product quality, reducing operational costs, and ensuring production safety. If industrial components with surface defects are applied in production or daily life, it can lead to irreversible safety issues and significant economic losses. Therefore, developing an efficient and accurate surface defect detection algorithm for industrial components is of great importance.

With the development of detection technologies, enterprises are continuously raising the quality standards for the surface of industrial components, and new methods are constantly being applied to surface defect detection of industrial components. Initially, traditional enterprises mainly relied on manual inspection techniques, which had the drawbacks of low sampling rates, high risks, and poor real-time performance. With the development of computer technology, computer vision-based detection methods have become the mainstream due to their high detection efficiency and low detection costs. Among them, non-deep learning-based object detection methods include four key steps: image preprocessing, target region selection, feature extraction, and classification. Gu et al. suggested an automatic inspection and recognition method for bearing surface defects using machine vision to detect and identify defects^1^. They adaptively improved the Canny algorithm based on the repetitive threshold segmentation technique and the Otsu algorithm, which enhanced the reliability and accuracy of bearing surface defect segmentation. Xu et al. proposed a method for detecting and recognizing the surface defects of Li-ion battery pole pieces based on multi-feature fusion and PSO-SVM^2^. Zhu et al. applied multidirectional Gabor filtering to the defective image, and the LBP features of the filtered image were combined with the 2DPCS feature histogram using the 2DPCA algorithm, which greatly improved the accuracy of metal surface defect detection^3^. Saadatmorad et al. proposed a series of damage detection methods based on wavelet transform, convolutional neural networks, and image processing techniques, which have been widely applied to crack identification and damage assessment in composite plates, steel beams, and historical masonry structures, providing important references for structural health monitoring^4–7^. However, although non-deep learning-based image processing methods can reduce the cost and improve the efficiency of manual inspection, issues such as difficult parameterization and poor applicability still prevent them from meeting industry needs.

In the field of computer vision, with the continuous progress of research on deep convolutional neural networks (CNN), the application of deep learning in defect detection has become increasingly widespread. The combination of deep learning and machine vision has significantly improved detection efficiency and the level of automation. Deep learning-based object detection methods are mainly divided into “two-stage” detection methods based on candidate regions and “one-stage” detection methods based on regression. Representative algorithms of the two-stage methods include R-CNN^8^, Fast R-CNN, and Faster R-CNN^9,10^. The one-stage detection algorithms have the advantage of faster execution speed, making them more suitable for industrial data detection. As a result, one-stage detection methods have become the dominant approach for industrial parts surface defect detection, with representative algorithms such as the YOLO series^11,12^. The YOLO algorithm outperforms other object detection algorithms due to its fast detection speed and the use of global context information, which enables real-time performance and reduces overlapping frame misdetections. Cheng et al. added an extra feature layer with small receptive fields to YOLOv3 and introduced the DIoU (Distance-IoU) loss function to enhance feature extraction and localization of small defects, addressing issues of defect omission and unclear features in small steel parts^13^. Guo et al. incorporated MobileNet-v3 into YOLOv4 to create a lightweight detection model, using it as the backbone network, and introduced the inverse residual structure and channel attention mechanism to improve defect detection accuracy^14^. Wang et al. proposed the YOLO-DWCSP-CA algorithm, based on the YOLOv5s network, specifically designed for surface defect detection in steel. This algorithm uses depth-separable convolution in the backbone network to enhance the acquisition of receptive fields^15^. Zhao et al. proposed the PC-YOLOv7 algorithm, based on the YOLOv7-tiny network, which replaces the ELAN structure in the backbone with the PC-ELAN structure. It also utilizes the bi-directional feature pyramid network (BIFPN) structure to promote the fusion of semantic and feature information, improving detection accuracy for small defects while reducing the number of parameters^16^. The YOLO series of models, as representative methods in the field of object detection, have continuously improved in speed, accuracy, and robustness with each iteration. Versions such as YOLOv5, YOLOv8, and YOLOv11 have been widely applied across various scenarios. However, despite the superior overall performance of newer versions, earlier versions like YOLOv5 still hold significant practical value. Specifically, the YOLOv5 architecture is lightweight and well-suited for embedded devices and resource-constrained environments, excelling in tasks that demand real-time performance. Additionally, YOLOv5 demonstrates more stable performance in specific scenarios, such as small object detection and target recognition in complex backgrounds, showcasing its unique advantages. Therefore, we believe that by making targeted improvements to the YOLOv5 architecture, its performance can be further enhanced for specific tasks and application scenarios.

Currently, existing defect detection algorithms suffer from low accuracy, as well as issues such as leakage and misdetection. This paper proposes a defect detection algorithm for industrial components based on an improved YOLOv5n, which enhances the recognition accuracy of defects on industrial component surfaces. The validity of the algorithm is verified on the NEU-DET dataset. A series of improvements and optimizations are made, using YOLOv5n as the baseline model.

1. The attention mechanism (CA) is introduced into the backbone network to enhance its feature extraction capability. The dynamic snake convolution is applied to transform the C3 module, allowing the model to better adapt to targets of varying scales, shapes, and structures. The dynamically changing convolution kernel captures target features more efficiently, enabling an adaptive sensory field and improving detection accuracy.
2. The Gold-YOLO structure replaces the neck path aggregation network (PANet) structure, retaining more gradual layer features, enhancing feature fusion, and improving detection accuracy.
3. The loss function is replaced with SIoU, whose angle loss and shape loss accelerate and improve the accuracy of anchor frame regression, enhance the flexibility of gradient assignment, and improve detection stability and robustness.

## YOLOv5n improved algorithm

**Fig. 1:**
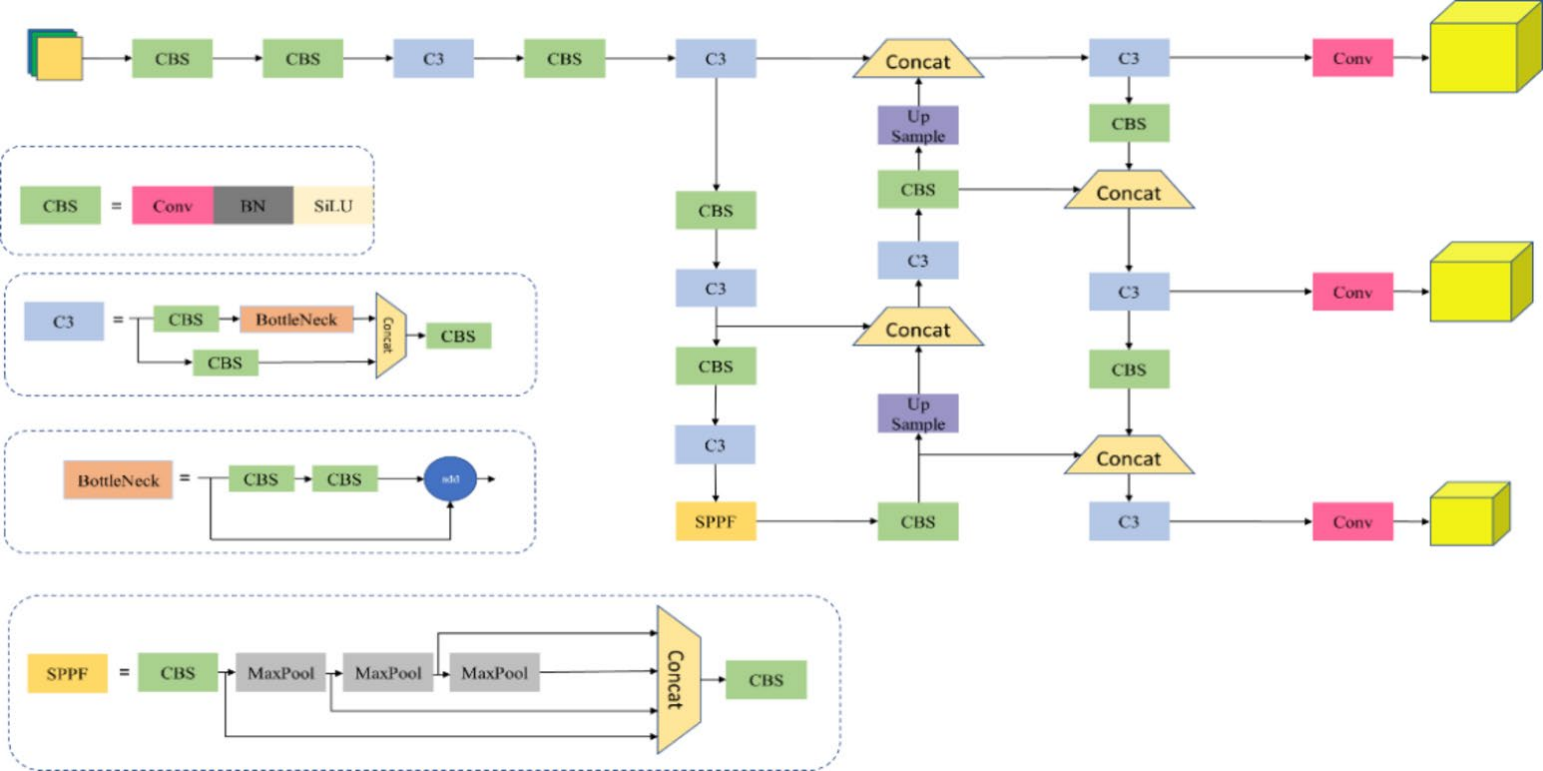
YOLOv5 network architecture diagram.

YOLOv5 is widely used in various target detection tasks. Its different versions mainly differ in network structure and parameter counts to meet different application scenarios and needs. YOLOv5n is typically used in scenarios requiring efficient real-time processing with relatively low accuracy requirements^17^. Compared to other YOLOv5 versions (YOLOv5s, YOLOv5m, YOLOv5l, YOLOv5x), YOLOv5n has fewer parameters and lower computational complexity, but with reduced accuracy, so it is often used in scenarios requiring efficient, real-time processing. Considering these factors, YOLOv5n is adopted as the baseline model for all improvements, with its structure shown in Fig. 1. The YOLOv5 model mainly consists of three parts: the backbone network, the neck network, and the head network.

### Gold-YOLO

**Fig. 2:**
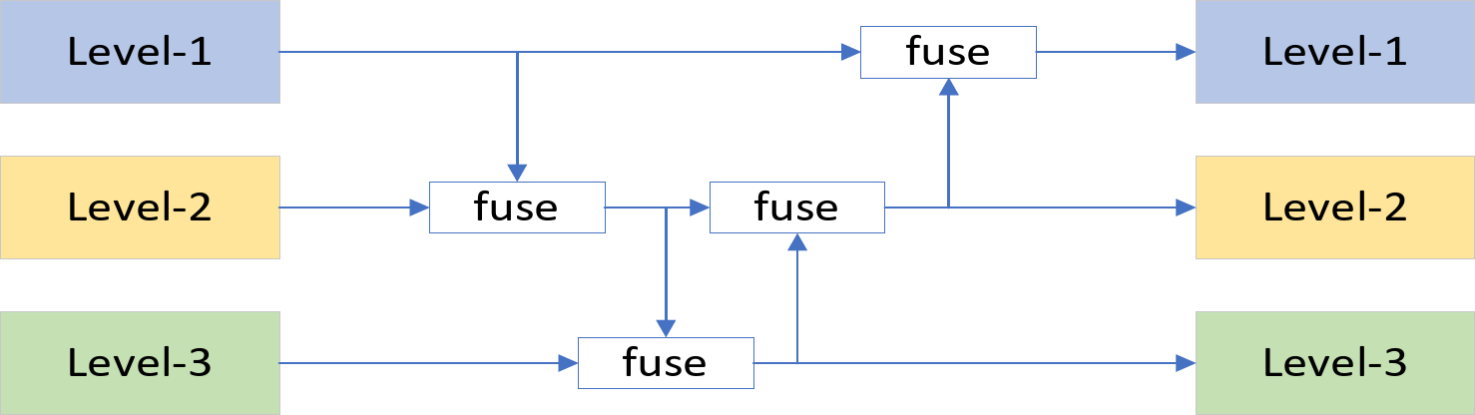
Neck structure of a conventional YOLO.

YOLOv5n is a lightweight version of the YOLO family, primarily designed for embedded devices and real-time applications. However, the original model suffers from information fusion issues. By integrating Gold-YOLO technology into YOLOv5n, the model’s multi-scale feature fusion capability is improved, significantly enhancing its performance in complex scenes while maintaining efficiency^18^. YOLOv5n’s neck structure uses a feature pyramid network (FPN) and its variants to fuse multi-level features, as shown in Fig. 2. However, YOLOv5n’s information fusion method has a notable flaw: when information needs to be integrated across layers, the traditional FPN structure cannot transfer information losslessly, which reduces the model’s information fusion capability. In contrast, GOLD-YOLO introduces a new collection and distribution mechanism that globally fuses multi-layer features and injects global information into higher layers. This mechanism greatly enhances the neck structure’s information fusion capability, improving the model’s overall performance.

Based on a global information fusion approach, TopFormer has achieved remarkable success in semantic segmentation tasks. GOLD-YOLO, derived from TopFormer’s theory, introduces a novel collection and distribution mechanism (GD) that enables efficient information exchange in YOLO by merging global features across levels and feeding global information to higher layers. This structure enhances information exchange in the neck without increasing latency, improving model recognition performance.

**Fig. 3:**
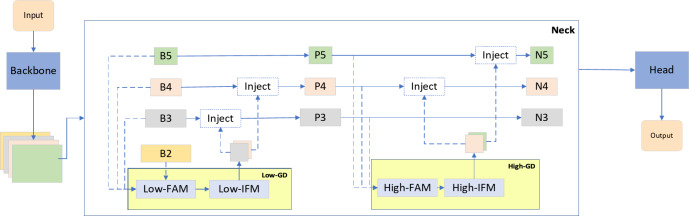
GOLD-YOLO structure.

GOLD-YOLO consists of three main modules: the Feature Alignment Module (FAM), Information Fusion Module (IFM), and Information Injection Module (Inject)^14^. First, the FAM aligns features from each level. Next, the IFM fuses the aligned features to generate global information. After obtaining the fused global information, the Inject module distributes it to each level using simple attentional manipulation to enhance detection. Two branches are used: the low-stage gather-and-distribute branch (Low-GD) and the high-stage gather-and-distribute branch (High-GD). These branches extract and fuse feature maps of different sizes through convolutional and attention-based blocks. The improved neck structure is shown in Fig. 3:

(1) FAM (Feature Attention Module)

The FAM module extracts feature from multiple levels of the backbone network (e.g., B2, B3, B4, B5) and performs alignment and normalization to unify the feature map sizes. This process ensures consistency of feature inputs while preserving key information across different scales, effectively reducing computational complexity.

1 $${F}_{align}=Low\_FAM\left(\left[B2,B3,B4,B5\right]\right)$$

Here, ${F}_{align}$ represents the aligned features processed by the FAM module, while B2, B3, B4, B5 are the feature outputs from different levels of the backbone network.

(2) IFM (Information Fusion Module)

The IFM module fuses the aligned features ${F}_{align}$ through convolution operations to generate unified global features and distributes the fused results to different levels. Through efficient convolution and feature splitting, IFM ensures the accurate transmission of features across different levels and the effective utilization of global information.

2 $${F}_{fuse}=RepBlock\left({F}_{align}\right)$$

3 $${F}_{inj\_p3},{F}_{inj\_p4}=Split\left({F}_{fuse}\right)$$

Here, ${F}_{fuse}$ represents the globally fused features, while ${F}_{inj\_p3}$ and ${F}_{inj\_p4}$ are the distributed features used for different detection layers.

(3) Inject (Injecting Module)

The Inject module fuses local features ${F}_{local}$ and global features ${F}_{inj}$. Using attention mechanisms, it dynamically adjusts the weights of the features, combining local embedding information to improve the detection accuracy of target features. Global activation feature computation:

4 $${F}_{global\_act\_P3}=resize\left(\sigma \left({Conv}_{act}\left({F}_{inj\_P3}\right)\right)\right)$$

Here, σ is the Sigmoid activation function, ${Conv}_{act}$ represents the convolution operation, and ${F}_{global\_act\_P3}$ is the weighted output of the global features. Fusion of local and global features:

5 $${F}_{att\_fuse\_P3}={Conv}_{local}\left({F}_{local}\right)\times {F}_{global\_act\_P3}+{F}_{global\_embed\_P3}$$

Here, ${F}_{att\_fuse\_P3}$ represents the fused feature map. Final feature update:

6 $$P3=RepBlock\left({F}_{att\_fuse\_P3}\right)$$

P3 is the output layer feature after injecting global features.

By introducing a joint attention mechanism that combines global and local information, the Inject module ensures precise information transfer during feature injection, enhancing the model’s adaptability to small targets and complex backgrounds.

In the Low-Stage Feature Alignment Module (Low-FAM), average pooling is used to under-sampling the input features, achieving uniformity in feature size. This technique ensures effective information aggregation while reducing computational complexity. The Inject module injects information at different levels through attention operations, with a RepBlock added after each attention fusion to further extract and refine the information. The High-Stage Feature Alignment Module (High-FAM) also uses average pooling to unify feature sizes, aiding in information aggregation and reducing the computational complexity of the transformer module. The High-Stage Information Fusion Module (High-IFM) consists of the transformer module and a splitting operation. The information injection module in the high-order clustering branch (High-GD) mirrors that in the low-order clustering branch (Low-GD). In this paper, the neck feature fusion structure of the YOLOv5n network is replaced with the GOLD-YOLO feature fusion structure to enhance the network’s fusion capability and improve detection accuracy.

### Backbone DSConv-CA module

**Fig. 4:**
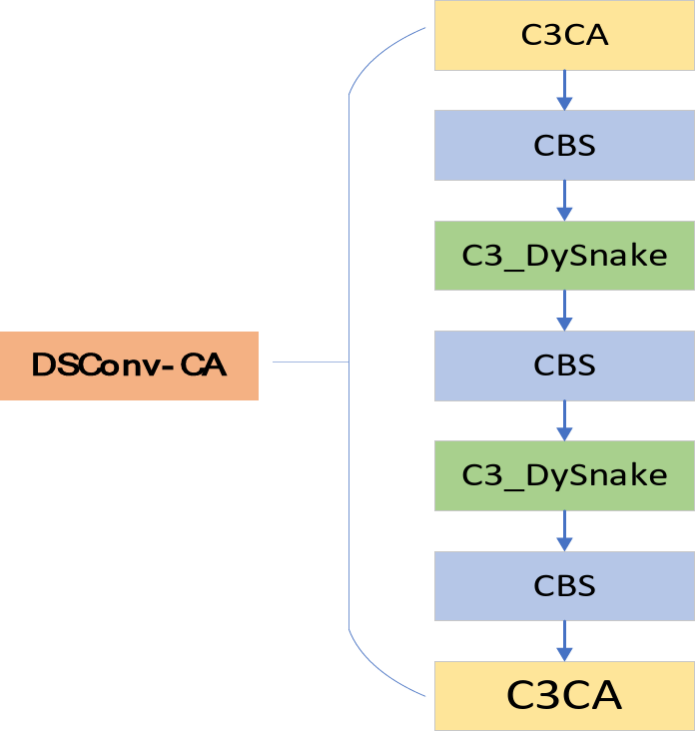
Improvement of YOLO backbone structure.

For detecting steel surface defects such as scratches and cracks, a backbone network is designed to better suit these defect types. The main improvements include the introduction of dynamic serpentine convolution and dynamic receptive field adjustment, which enhance the ability of convolutional neural networks (CNN) to detect complex object shapes and dynamic contours, thereby improving detection of long, strip-shaped defects. The Attention Mechanism Module, C3CA, is introduced to significantly enhance feature extraction by combining Cross-Stage Cascade and Channel Attention. The block diagram of the improved system is shown in Fig. 4:

#### Attention mechanism module C3CA

Attention mechanisms have been highly successful in various computer vision tasks, including image classification, target detection, semantic segmentation, video understanding, 3D vision, multimodal tasks, and semi-supervised learning^19^. They are typically categorized as follows: channel attention, spatial attention, combined channel-spatial attention, temporal attention, combined spatial-temporal attention, and branching attention mechanisms. Each mechanism focuses on different aspects of target features.

**Fig. 5:**
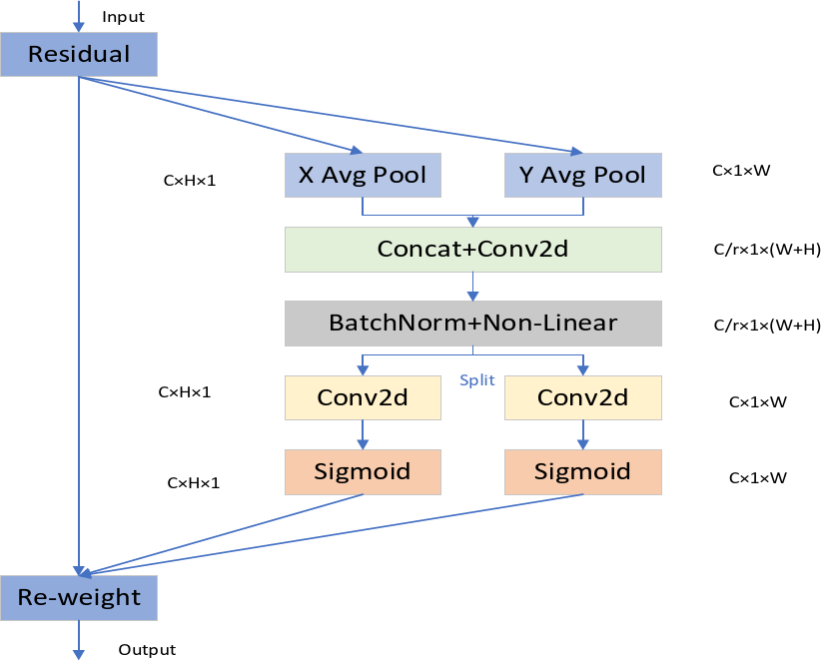
CA attention mechanism.

Compared to previous methods of attention mechanisms, Coordinate Attention (CA) has the following advantages: CA is not only able to capture cross-channel information, but also direction-aware and position-aware information, which can effectively help the model to establish more accurate localization and identify the target of interest; secondly, CA has a very small number of parameters that can be flexibly embedded into the network and feature extraction structure; finally CA has a very powerful scalability that can be easily applied to computer vision downstream tasks such as image classification and instance segmentation, etc. The structure of CA attention mechanism is shown schematically in Fig. 5.

The C3 module in the YOLO algorithm contains three convolutional blocks, and the core part is a stack of Bottleneck modules. By integrating the CA mechanism in Bottleneck, the characterization ability of feature channels and spatial information can be enhanced. The specific steps are as follows:

**Step 1: Channel feature encoding**.

For the input data of channel (C) × height (H) × width (W), the CA attention mechanism first encodes the channel through two average pooling operations, and the output expression of the Cth channel with height H and width W is shown as follows:

7 $$\left\{\begin{array}{c}{Z}_{c}^{h}\left(h\right)=\frac{1}{W}\sum _{0\le i\le W}{x}_{c}\left(h,i\right)\\ {Z}_{c}^{w}\left(w\right)=\frac{1}{H}\sum _{0\le j\le H}{x}_{c}\left(j,w\right)\end{array}\right.$$

In the formula H, W denote the height and width of the Cth channel feature map, respectively, by aggregating the information in the direction of width and height dimensions, the global information at the channel level is extracted from the height and width directions, respectively, which helps to help the network to locate the target of perceptual interest more accurately.

**Step 2: Channel splicing and convolution**.

The features ${Z}^{h}$ in the height direction and ${Z}^{w}$ in the width direction are concatenated in the channel dimension to form a new feature representation:

8 $${Z}_{concat}=[{Z}^{h},{ Z}^{w}]$$

Subsequently, the spliced features are downscaled and smoothed by a 1 × 1 convolutional layer F1:

9 $$f={\updelta }\left({F}_{1}\left[{Z}^{h},{Z}^{w}\right]\right)$$

Here, δ is the activation function, and ${F}_{1}$ is a shared 1 × 1 convolution operation used to generate the fused feature representation $f$. The function of this step is to fuse the information in the height and width directions to form the feature representation with a global receptive field.

**Step 3: Generate attentional weights**.

Attentional weights (height weights and width weights) for both directions are generated from $f$. Height direction weight ${g}^{h}$: For the height direction information in $f$, it is obtained by an independent 1 × 1 convolution and nonlinear mapping:

10 $${g}^{h}=\sigma ({W}_{h}\times {f}_{h})$$

${W}_{h}$ is the convolutional weight in the height direction and σ is the Sigmoid function.

Width direction weight ${g}^{w}$: for the width direction information in $f$, obtained by another independent 1 × 1 convolution and nonlinear mapping:

11 $${g}^{w}=\sigma ({W}_{w}\times {f}_{w})$$

${W}_{w}$ is the convolution weight in the width direction. The ${g}^{h}$ and ${g}^{w}$ generated in this step represent the attentional weights of the channel in the height and width directions.

**Step 4: Attention weight expansion and weighting**

Extend the attention weights in the height and width directions to the same size as the input feature maps and act on the input feature maps respectively: height direction attention weighting:

12 $${y}_{c}\left(i,j\right)={x}_{c}\left(i,j\right)\times {g}_{c}^{h}\left(i\right)$$

Here, ${g}_{c}^{h}\left(i\right)$ is an extended version of the height-direction attentional weighting, denoting the height weight of the cth channel. Width direction attention weighting:

13 $${y}_{c}\left(i,j\right)={y}_{c}\left(i,j\right)\times {g}_{c}^{w}\left(j\right)$$

Here, ${g}_{c}^{w}\left(j\right)$ is an extended version of the width direction attention weighting, denoting the width weight of the cth channel. The final output is:

14 $${F}_{out}(c,i,j)={F}_{in}(c,i,j)\times {g}_{c}^{h}\left(i\right)\times {g}_{c}^{w}\left(j\right)$$

This step completes the weighting of the attentional weights and encodes the information in the height and width directions into the input features to obtain the enhanced features.

**Step 5: Integration into C3 module**

For Bottleneck in C3 module, extract the main branch feature ${F}_{bottleneck}$, input ${F}_{bottleneck}$ to CA module, get the enhanced feature ${F}_{CA}=CA\left({F}_{bottleneck}\right)$and splice it with the residual branch feature ${F}_{residual}$:

15 $${F}_{out}=Concat({F}_{CA},{F}_{residual})$$

and the final output ${F}_{out}$ is the output of the C3 module after integrating CA attention.

The C3CA module is an enhanced convolutional block that helps the model focus more on important feature channels, thereby improving its detection performance. By introducing the attention mechanism, the C3CA module can dynamically learn the importance of different channels in the feature map to better capture the key features of the target. This significantly enhances the feature extraction capability of convolutional neural networks and further improves detection accuracy. In YOLOv5n, the introduction of the C3CA module enhances feature extraction capabilities, improves gradient transfer, and optimizes multi-scale feature fusion, achieving a better balance between lightweight design and high performance. Compared to traditional convolutional operations, the C3CA module does not significantly increase computational overhead, making it capable of improving model performance while maintaining computational efficiency.

#### Dynamic snake Convolution

The YOLO algorithm often struggles to detect steel surface defects with complex shapes and varying orientations due to the fixed geometric transformations of its convolutional kernels. Inspired by active contour models, we integrate the Dynamic Snake Convolution layer into YOLO’s C3 module to significantly enhance feature extraction, adapt to multi-scale targets, and improve detection accuracy, especially for irregular, elongated defects. Traditional convolution operations apply the same kernel within a fixed window, with a static, uniform grid structure in 2D space, limiting the network’s ability to adapt to complex, varying shapes. Inspired by deformable convolutions, DSConv introduces deformable offsets to traditional convolutions. To prevent the model from learning deformable offsets freely, which could lead to deviations in the receptive field, DSConv employs an iterative strategy. The position of each convolutional operation is determined by using all deformable offsets concerning the central grid as a reference, ensuring the continuity of attention^20^. By adding continuity constraints to the kernel design, DSConv allows the kernel to “walk” across the input feature map in a non-rigid, deformable manner, forming curved or stretched shapes to better adapt to complex structures in the image. Each convolution position is based on the previous one, freely choosing the direction while maintaining the continuity of the receptive field. Active contour models dynamically adjust their shape to match object boundaries, demonstrating strong adaptability to complex geometries^21^.

The specific process is as follows: local feature extraction of the tubular structure is performed by dynamic serpentine convolution (DSConv), assuming that the given convolution center coordinates are$\text{K}\text{i} = (\text{x}\text{i} , \text{y}\text{i})$ A is a 3 × 3 convolution kernel, then K can be denoted as

16 $$K=\left(X-1,Y-1\right)\cdot \left(Y-1,Y\right),\ldots,\left(X+1,Y+1\right)$$

In order to provide greater focusing flexibility for the convolution kernel, deformation offsets ∆ are introduced, and deformation offsets can be learned freely under a thin tubular structure. conceptual fields tend to undergo target offsets, so an iterative strategy is used to observe the to-be-processed targets in sequence, thus ensuring continuity of attention without distracting from the large to small deformation offsets. A convolution kernel of size 9 is selected in DSConv, and the position information of each convolution kernel in terms of the X-axis for example is expressed as ${K}_{i}\pm c = ({x}_{i}\pm c,{ y}_{i}\pm c)$, where $C=\left\{0,\,1,\,2,\,3,\,4\right\}$, the position from the center ${K}_{i}$, position away from the center grid ${K}_{i}+1$ In contrast to this, an offset is added $\varDelta = \left\{\delta \right|\delta \in [-1, 1]\}$, thus in order to ensure that the offset of the convolution kernel $\varSigma$ conforms to a linear form.

Expression on the X-axis and on the Y-axis, respectively:

17 $$\left\{\begin{array}{c}{K}_{i\pm c}=\left\{\begin{array}{c}\left({X}_{i+c},{Y}_{i+c}\right)=({X}_{i}+c,{Y}_{i}+\sum_{i}^{i+c}\varDelta y)\\ \left({X}_{i-c},{Y}_{i-c}\right)=({X}_{i}-c,{ Y}_{i}+\sum _{i-c}^{i}\varDelta y)\end{array}\right.\\ {K}_{j\pm c}=\left\{\begin{array}{c}\left({X}_{j+c},{Y}_{j+c}\right)=({X}_{j},\sum _{j}^{j+c}{ Y}_{j}+c)\\ \left({X}_{j-c},{Y}_{j-c}\right)=({X}_{j},\sum_{j}^{j+c}{Y}_{j}-c)\end{array}\right.\end{array}\right.$$

Since offsets are usually fractional, bilinear interpolation is implemented as:

18 $$K=\sum_{{K}^{{\prime}}}B\left({K}^{{\prime}}\cdot K\right)\cdot {K}^{{\prime}}$$

where$\text{K}$ denotes the fractional position of Eq. (2), and ${K}^{{\prime}}$ is the enumeration of all complete spatial locations, and $\text{B}$ is the bilinear interpolation kernel divided into two one-dimensional kernels:

19 $$B\left({K\cdot K}^{{\prime}}\right)=b\left({K}_{x},{K}_{x}^{{\prime}}\right)\cdot b\left({K}_{y},{K}_{y}^{{\prime}}\right)$$

**Fig. 6:**
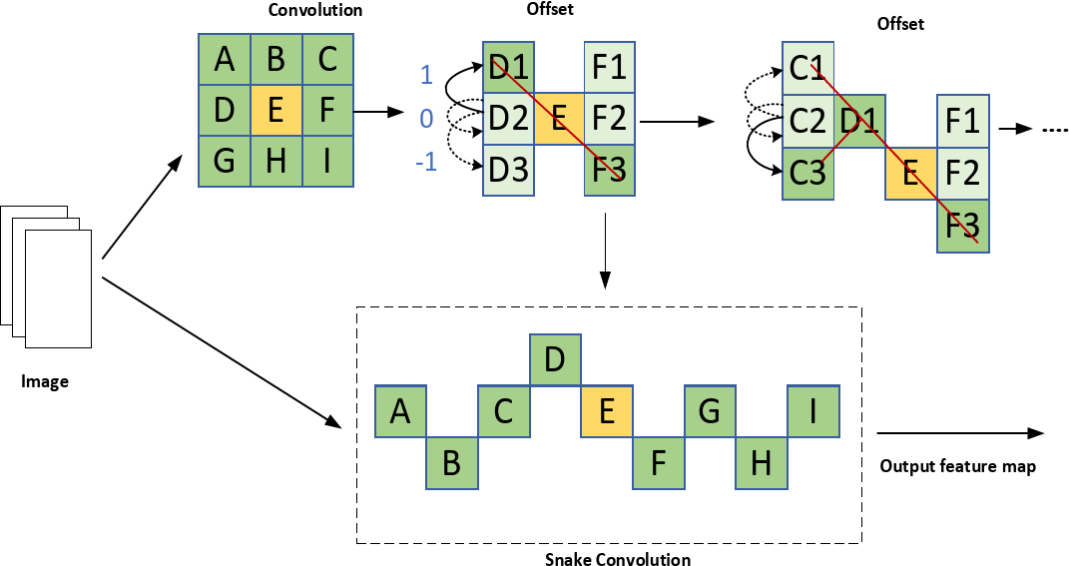
Dynamic serpentine convolution.

In order to make the convolutional kernel more adaptable to the complex geometric features of the image, deformation offsets are introduced. The model learns the deformation offsets randomly, then the perceptual region may deviate from the target, and the iterative strategy is shown in Fig. 6. The strategy sequentially matches each target with an observable location, ensuring continuous feature attention without over-dispersing the perceptual region with large deformation offsets.

**Fig. 7:**
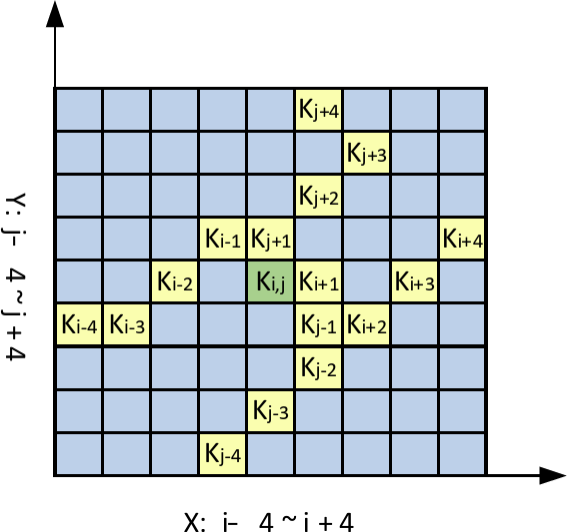
DSConv sensory field change map.

DSConv is a deformed 2D transformation process covering 9 × 9, which enhances the ability to capture key features, such as in industrial part surface defect detection, where targets may be distant, small, or slender. As shown in Fig. 7, DSConv spans a 9 × 9 area during deformation, expanding the model’s receptive field and improving the perception of key features, enabling more accurate target recognition. The introduction of dynamic serpentine convolution allows adaptive adjustment of the convolution kernel shape, capturing local image features more precisely and improving the handling of complex images. This enhances feature extraction capability and model robustness. By combining conventional convolution with dynamic serpentine convolution, the stability and efficiency of traditional convolutions are preserved, while introducing the flexibility and adaptability of DSConv.

Dynamic snake convolution optimizes the convolution kernel’s offset through a learning process, enabling adaptive path adjustments to better match complex shapes in the input feature map. This enhances the network’s ability to recognize and extract non-regular shapes, particularly for locally elongated, highly varied zigzag structures, resulting in significant performance improvement. By introducing dynamic serpentine convolution, the model’s capacity to handle complex geometric information is enhanced. With this deformable convolution, the network more effectively captures target defects, further boosting the model’s generalization ability and recognition accuracy.

### SIOU loss function

The aspect ratio of the predicted frame to the real frame, the overlap region and other target frame metrics are the basis for the establishment of conventional target recognition loss functions, such as GIoU, DIoU, CIoU^22^. The original model yolov5 adopts the CIoU as a loss function instead of the traditional IoU, which combines several factors such as IoU, centroid distance, and the bounding box aspect ratio, which improves the detection performance to some extent. However, CIoU also has some drawbacks, such as higher computational complexity, limited effect on small target detection, and its introduction of aspect ratio has some irrationality, which cannot take into account the direction of regression each time, resulting in slow regression speed, and the adaptability of CIoU to different scenarios and datasets varies, and in some specific scenarios, CIoU may not be significantly better than the other loss functions, the It may even perform worse.

Therefore, in order to accelerate the convergence speed of the model and make the model more suitable for small target detection, we adopt the SIoU loss function. The SIoU loss function proposed by Gevorgyan further takes into account the vector angle between the prediction frame and the real frame, and integrates the position and category information of the target, so that the model can evaluate the accuracy of the detection results in a more comprehensive way^23,24^. The SIoU consists of four parts: angle cost, distance cost, shape cost, and IoU cost.

(1) Angular loss

**Fig. 8:**
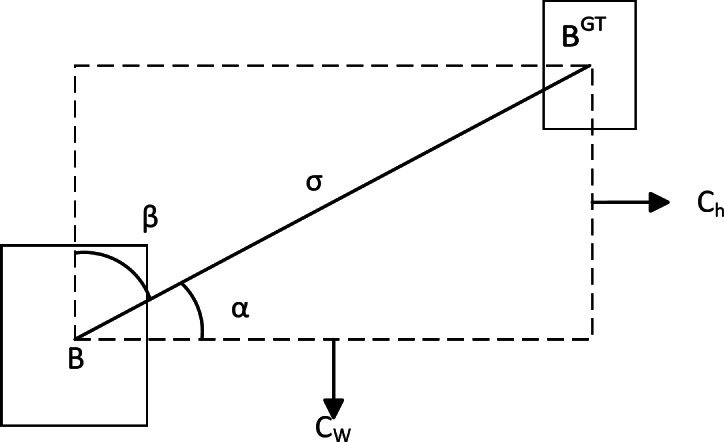
Angle cost diagram of the SIoU loss function.

Angular loss is defined as follows and the schematic diagram is shown in Fig. 8.

20 $$\left\{\begin{array}{c}\varLambda=1-2*{sin}^{2}\left(arcsin\left(\frac{{C}_{h}}{\sigma}\right)-\frac{\pi}{4}\right)\\{C}_{h}=max\left({b}_{{c}_{y}}^{gt},{b}_{{c}_{y}}\right)-min\left({b}_{{c}_{y}}^{gt},{b}_{{c}_{y}}\right)\\\sigma=\sqrt{{\left({b}_{{c}_{x}}^{gt}-{b}_{{c}_{x}}\right)}^{2}+{\left({b}_{{c}_{y}}^{gt}-{b}_{{c}_{y}}\right)}^{2}}\end{array}\right.$$

where.${C}_{h}$ is the height difference between the center point of the real frame and the predicted frame, and${\upsigma}$ is the distance between the center point of the real frame and the predicted frame, and${b}_{{c}_{x}}^{gt},{b}_{{c}_{y}}^{gt}$ are the coordinates of the center of the real frame, ${b}_{{c}_{x}},{b}_{{c}_{y}}$ are the coordinates of the center of the prediction frame.

(2) Distance loss

**Fig. 9:**
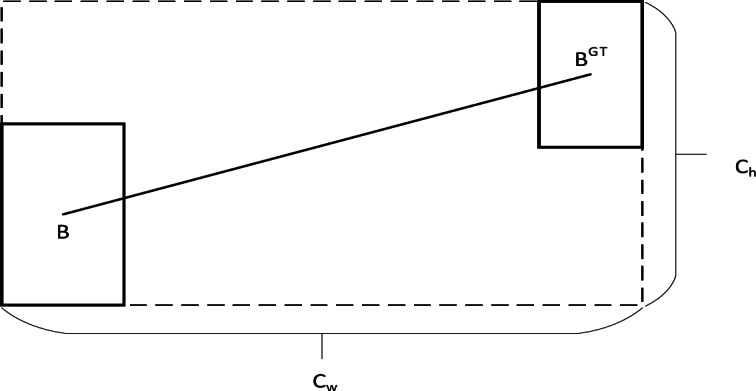
Distance cost diagram of the SIoU loss function.

The distance loss is related to the minimum outer rectangle of the true and predicted frames and is defined as follows and the schematic diagram is shown in Fig. 9:

21 $$\left\{\begin{array}{c}\varDelta=\sum_{t=x,y}\left(1-{e}^{-\gamma{\rho}_{t}}\right)=2-{e}^{-\gamma{\rho}_{x}}-{e}^{-\gamma{\rho}_{y}}\\{\rho}_{x}={\left(\frac{{b}_{{c}_{x}}^{gt}-{b}_{{c}_{x}}}{{C}_{w}}\right)}^{2}\\{\rho}_{y}={\left(\frac{{b}_{{c}_{y}}^{gt}-{b}_{{c}_{y}}}{{C}_{h}}\right)}^{2}\\\gamma=2-\varLambda\end{array}\right.$$

${C}_{w}$ and ${C}_{h}$ are the width and height of the smallest outer rectangle of the real and predicted boxes.

(3) Shape loss

Shape loss is defined as follows

22 $$\left\{\begin{array}{c}\varOmega =\sum _{t=w,h}{\left(1-{e}^{-{w}_{t}}\right)}^{\theta }={\left(1-{e}^{-{w}_{w}}\right)}^{\theta }+{\left(1-{e}^{-{w}_{h}}\right)}^{\theta }\\ {w}_{w}=\frac{\left|w-{w}^{gt}\right|}{max\left(w,{w}^{gt}\right)}\\ {w}_{h}=\frac{\left|h-{h}^{gt}\right|}{max\left(h,{h}^{gt}\right)}\end{array}\right.$$

w, h, ${w}^{gt}$, ${h}^{gt}$ being the width and height of the predicted and real boxes, ${\uptheta }$ is the degree of attention to shape loss.

(4) IoU losses

IoU is the intersection and concurrency ratio between the prediction frame (Pre) and the true frame (GT). The IoU is defined as follows:

23 $$\text{I}\text{o}\text{U}=\frac{{P}_{re}\cap GT}{{P}_{re}\cup GT}$$

In summary, the final SIoU loss equation is.

24 $${\text{L}\text{o}\text{s}\text{s}}_{SIoU}=1-\text{I}\text{o}\text{U}+\frac{\varDelta +{\Omega }}{2}$$

## Experimental results and analysis

### Data sets

**Fig. 10:**
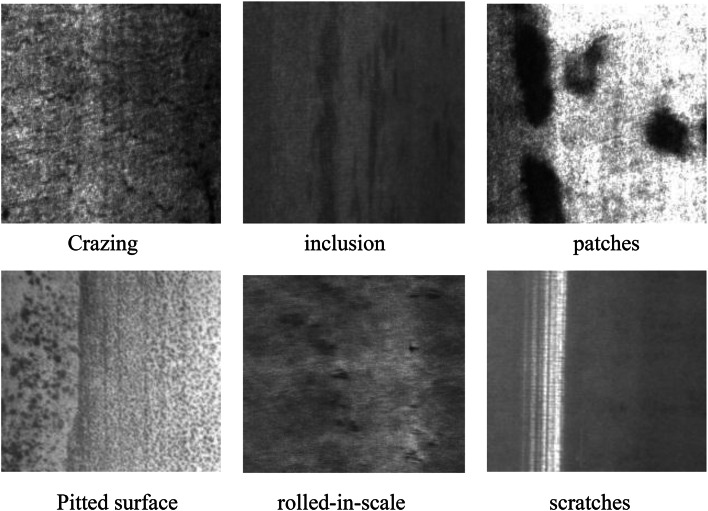
Images of selected datasets.

The dataset employed in the experiments for this paper is the NEU-DET dataset, which is a surface defect database provided by Northeastern University. It comprises six defect categories: Crazing (CR), Inclusion (IN), Patches (PA), Pitted Surface (PS), Rolled-in Scale (RS), and Scratches (SC). Each category contains 600 samples, with a total of 1,800 images. A selection of the dataset images is presented in Fig. 10.

Scratches are linear marks that may appear on a surface when it is slid over a hard object during the production or handling of an industrial part. Patches are irregular areas with variations in surface color or material, possibly due to uneven material composition. Cracks usually appear as microscopic cracks in the surface of the material. Inclusions are the presence of foreign matter, such as grit or other oxides, on the surface or within the material. Pitting surfaces are small depressions on the surface of the material, usually caused by corrosion or physical damage to the material, which may be confused with the texture of the material itself during inspection, especially if the material itself has a rough or uneven surface. Rolled-in layers are oxidized layers that are pressed into the surface of the material during the hot rolling process. These defects are constantly changing in shape, and some of them are very similar to the color and texture of the material surface, which may lead to misdetection or omission during inspection, thus affecting the inspection efficiency.

The distribution of each category in the training dataset is shown in Table 1, and data augmentation as well as label smoothing strategies are used in the training process. The model training is set to 200 epochs and the size of batch-size is 16, keeping these basic parameters unchanged, using YOLOv5n as the baseline model, gradually adding the improvement scheme for training and testing, and comparing and analyzing with some of the current mainstream models in order to evaluate the parameters and performance of each model.

**Table 1:** Distribution of categories in the training dataset.

Form	CR	IN	PA	PS	RS	SC
Amount	538	797	702	339	507	426

### Algorithm evaluation metrics

To evaluate the model parameters and detection criteria, average precision (AP) is utilized as the metric for each defect category, while mean average precision (mAP) and the number of model parameters are applied to assess the overall performance of the network. The specific formulas for AP and mAP are as follows:

25 $$\left\{\begin{array}{c}P=\frac{TP}{TP+FP}\\ R=\frac{TP}{TP+FN}\\ AP={\int }_{0}^{1}P\left(R\right)dR\\ mAP=\frac{1}{N}\sum _{i=1}^{N}A{P}_{i}\end{array}\right.$$

P and R represent precision and recall, respectively, while N denotes the number of categories. TP refers to instances where the target was fully and accurately detected, FP represents cases where an incorrect sample was mistakenly identified as correct by the model (false positive), and FN indicates that a positive target was incorrectly classified as negative (missed detection).

### Comparison algorithm and experimental environment

The software and hardware configuration of the experimental environment in this paper is shown in Table 2.

**Table 2:** Software and hardware configuration of the experimental environment.

Name (of a thing)	Configuration parameters
Operating system	26-22.04.1-Ubuntux86 64-GNU/Linux
Deep Learning Framework	Python-3.9.12 torch-2.1.0 + cu121
Compilation software	Pycharm
CPU	Intel(R)Xeon(R)-Gold-5218R-CPU-@-2.10 GHz
GPU	Nvidia’Tesla: A40.48G

#### Ablation experiments of integrating the C3-DSConv module

During the training process, we used an adaptive loss function that dynamically adjusts the model’s weights based on changes in the input data during each iteration. In addition, we employed a stepwise optimization strategy, allowing the model to gradually adapt to different input data. Compared to traditional convolution operations, the dynamic serpentine convolution can adjust the position of the convolution kernel according to the dynamic changes in the data, thereby extracting features more accurately. This flexible adjustment mechanism provides a significant advantage when dealing with complex data. We also compared the performance of traditional convolution methods with dynamic serpentine convolution during the training process, and the results showed that dynamic serpentine convolution significantly improves the model’s accuracy while maintaining computational efficiency.

An ablation experiment was conducted on the test set to verify the effectiveness of the algorithm and the capability of the C3-DSConv module in detecting elongated and curved defects on the surface of industrial aluminum plates. To ensure the validity of the experiment, the experimental environment and parameter settings were kept consistent with the previous section. The corresponding results are reported in Table 3.

**Table 3:** Performance comparison of models using C3 and C3-DSConv.

Method	AP%						mAP@50 (%)	Params (M)
	CR	IN	PA	PS	RS	SC		
C3	35.8	85.8	80.8	85.5	51.6	86.4	71	1.76
C3-DSConv	36.8	86.9	81	86.2	53.2	88.4	72.1	2.01

From the experimental results in Table 3, it can be observed that the inclusion of the C3-DSConv module significantly improves the detection accuracy for various types of defects. Compared to the baseline model, the accuracy rates for cracks, inclusions, patches, pitting, rolled oxide scales, and scratches increased by 1.0%, 1.1%, 0.2%, 0.7%, 1.6%, and 2.0%, respectively. Notably, the improvement in scratch detection is the most significant, which is particularly important as scratches on industrial aluminum plates typically manifest as irregular, elongated, and curved shapes. These elongated structures occupy a relatively small portion of the image and have limited pixel representation. Therefore, the experimental results demonstrate that integrating the DSConv into the C3 module can effectively enhance the model’s ability to detect elongated and subtle defects.

#### C3CA attention experiment

To validate the effectiveness of the improved attention in this paper, attention mechanisms such as SE, CBAM, ECA, and CA were selected to be added into the baseline YOLOv5n model for the detection performance comparison respectively, and the performance results are shown in Table 4.

**Table 4:** Model performance effect using YOLOv5n and adding C3CA, C3ECA, C3CBAM, C3SE respectively. Significant values are in bold.

Method	AP (%)						mAP@50 (%)	Params (M)
	CR	IN	PA	PS	RS	SC		
YOLOv5n	35.8	85.8	80.8	85.5	51.6	86.4	71	1.76
YOLOv5n + C3CA	37.3	87.9	80.3	83.3	**56.8**	**89.9**	**72.5**	1.86
YOLOv5n + C3ECA	**40.2**	87.4	**81.1**	86.3	55.2	87.4	72.2	1.76
YOLOv5n + C3CBAM	39.6	87.8	77.4	**87.8**	45.8	85	71.4	1.8
YOLOv5n + C3SE	37	**91.8**	78.2	82.5	52	85.1	71.6	**1.69**

From Table 3, it can be seen that the original model of YOLOv5n has a mAP of 71%, which is increased after the addition of the channel attention mechanism. Using the attention mechanism C3CA module, the mAP is raised to 72.5%, which is 1.5% higher than the original module; the model uses the C3ECA attention mechanism, which raises the mAP to 72.2%, which is 1.2% higher than the original model. The other two attention mechanisms, C3CBAM and C3SE, improved less, 0.4% and 0.6%, respectively. Therefore, after comprehensive consideration, we chose to introduce the C3CA attention mechanism module into the YOLOv5n model, which improved the model performance to a certain extent after its introduction.

#### Comparison experiment on loss functions

In this study, we conducted a comparative experiment on the performance of CIoU and SIoU as regression loss functions to evaluate their effectiveness in the task of defect detection on industrial component surfaces. The experimental results show that the model using SIoU as the loss function outperforms the one using CIoU in terms of both detection accuracy and stability. Specifically, CIoU optimizes the position of the bounding box by considering the distance between the center points, the overlap area, and the aspect ratio consistency. However, its flexibility in gradient distribution is relatively low, particularly in cases where the target box is rotated or has a complex shape, which may lead to a slower optimization process. In contrast, SIoU introduces angle loss and shape loss in its design, enabling the bounding box to approach the ground truth box more quickly while exhibiting stronger adaptability to rotation and shape variations during the optimization process. This design effectively addresses the localization bias caused by complex shapes or elongated targets commonly found in industrial defects. The specific experimental data are shown in Table 5:

**Table 5:** Performance comparison of models using CIoU and SIoU.

Loss function	AP (%)						mAP@50 (%)	Params (M)
	CR	IN	PA	PS	RS	SC		
CIOU	35.8	85.8	80.8	85.5	51.6	86.4	71	1.76
SIOU	38.7	88.2	83.8	80.1	52.9	85.5	71.5	1.76

Based on the experimental data, the model using SIoU demonstrated a 2.1% improvement in mAP on the NEU-DET dataset, with particularly notable performance in the detection of elongated and small target defects. Additionally, the gradient distribution of SIoU is more uniform, which helps enhance the model’s convergence speed and detection robustness.

#### Ablation experiments

In order to verify the effectiveness of the three improvement schemes proposed in this paper, a set of ablation experiments were designed on the NEU-DET dataset with the YOLOv5n network model as the baseline, and the environment and parameter settings were kept unchanged during the realization process, as shown in Table 6.

**Table 6:** Ablation experiments for the improved program. Significant values are in bold.

Backbone	Neck	SIoU	AP (%)						mAP@50 (%)	Params (M)
			CR	IN	PA	PS	RS	SC		
			35.8	85.8	80.8	85.5	51.6	86.4	71	1.76
✓			37.5	88.4	80.8	84.7	53.3	89.4	72.5	2.01
	✓		38.6	89.4	83.3	85.3	53	88.6	72.8	4.64
		✓	38.7	88.2	83.8	80.1	52.9	85.5	71.5	1.76
✓	✓		39.5	90.2	81.6	82.7	54.8	87.4	73.2	4.89
✓		✓	43.1	87.4	83.2	81.3	56.4	86.9	73	2.01
	✓	✓	34.7	87.1	**85.6**	84.3	**61.2**	89.3	74	4.64
✓	✓	✓	**50.4**	**90.5**	84.5	**86.1**	54.3	**89.7**	**75.3**	4.89

As can be seen from the table, in the single improvement scheme, the improvement of the backbone module increases the mean value of the average accuracy of the baseline model by 1.5%, which indicates that after the improvement of the backbone module, the sensory field is expanded and the model performance is improved, and after the introduction of the dynamic serpentine convolution, the model can more accurately detect the scratches, cracks and other types of defects in the surface defects of the industrial parts; the introduction of gold-yolo neck structure improves the average detection accuracy of the model by 1.8%, which shows that the information fusion ability of the model is enhanced after the improvement, which improves the feature extraction ability of the model and thus improves the detection accuracy of the model; after replacing the loss function with the SIoU, the average detection accuracy of the model improves by 0.5%, which shows that the angular loss and shape loss introduced by the SIoU loss function can make the anchor frame faster and more accurate in the regression process, thus improving the average detection accuracy of the model. From the data obtained in the ablation experiments, it is found that the mAP of the defect categories obtained from the final improved model is the highest, except for the mAP of the categories of patches and Rolled-in Scale, which is slightly lower than that of the category improved by adding Neck and SIoU only, and the rest of the defect categories are the highest. In particular, the Crazing and Inclusion defect categories have the most significant improvement in mAP, which results in the best model performance and the highest overall average detection accuracy.

#### Performance comparison of different algorithmic models

In order to further evaluate the performance of the improved algorithm in this paper, several mainstream target detection algorithms, such as SSD, YOLOv3, YOLOv7, YOLOV8s and other different algorithms and the improved algorithm of this paper are selected for the comparison of the detection performance under the benchmark of the same dataset division, and the specific results are shown in Table 7.

According to Table 7, the improved model in this paper achieves a mean average precision superior to other mainstream detection algorithms. Compared to the SSD model, it shows a 15.2% improvement; compared to the YOLOv3 model, it achieves a 7.1% increase; compared to the YOLOv9-t model, it improves by 1.2%; and compared to the PP-YOLOE model, it demonstrates a 3.2% enhancement. The detection results indicate that current detection algorithms exhibit relatively low precision for detecting Crazing defects in the NEU-DET dataset. However, the improved algorithm achieves the most significant precision improvement for detecting Crazing defects.

In summary, compared to other models, the improved model performs significantly better in most categories, such as CR, IN, PA, and SC, showcasing its outstanding generalization ability in diverse scenarios.

**Table 7:** Performance comparison of different detection algorithms.

Method	AP (%)						mAP@50 (%)
	CR	IN	PA	PS	RS	SC	
SSD	28	73.1	83.4	73.5	54.3	33.1	60.1
Faster R-CNN	35.7	80.6	79.8	84.6	52.6	85.3	70.5
YOLOv3	28.3	75	82.4	80.6	52.1	88.3	68.2
YOLOv4	36.5	76.8	81.8	83.2	52.9	85.9	70.6
YOLOv5n	35.8	85.8	80.8	85.5	51.6	86.4	71
YOLOv7	35.6	83.5	81	86	53.2	86.5	71.3
YOLOV8s	38.2	86.9	80.4	82.6	54	88.6	72.3
YOLOv9-t	42.3	89.5	82.1	88.3	53.1	89.1	74.1
YOLOv10n	36.4	85.1	80.6	83.5	53.4	88.5	72.4
PP-YOLOE-s	37.8	84.2	79.6	84.4	52.4	87.2	72.1
Proposed algorithm	50.4	90.5	84.5	86.1	54.3	89.7	75.3

#### Visualization analysis

**Single defect**.

**Fig. 11:**
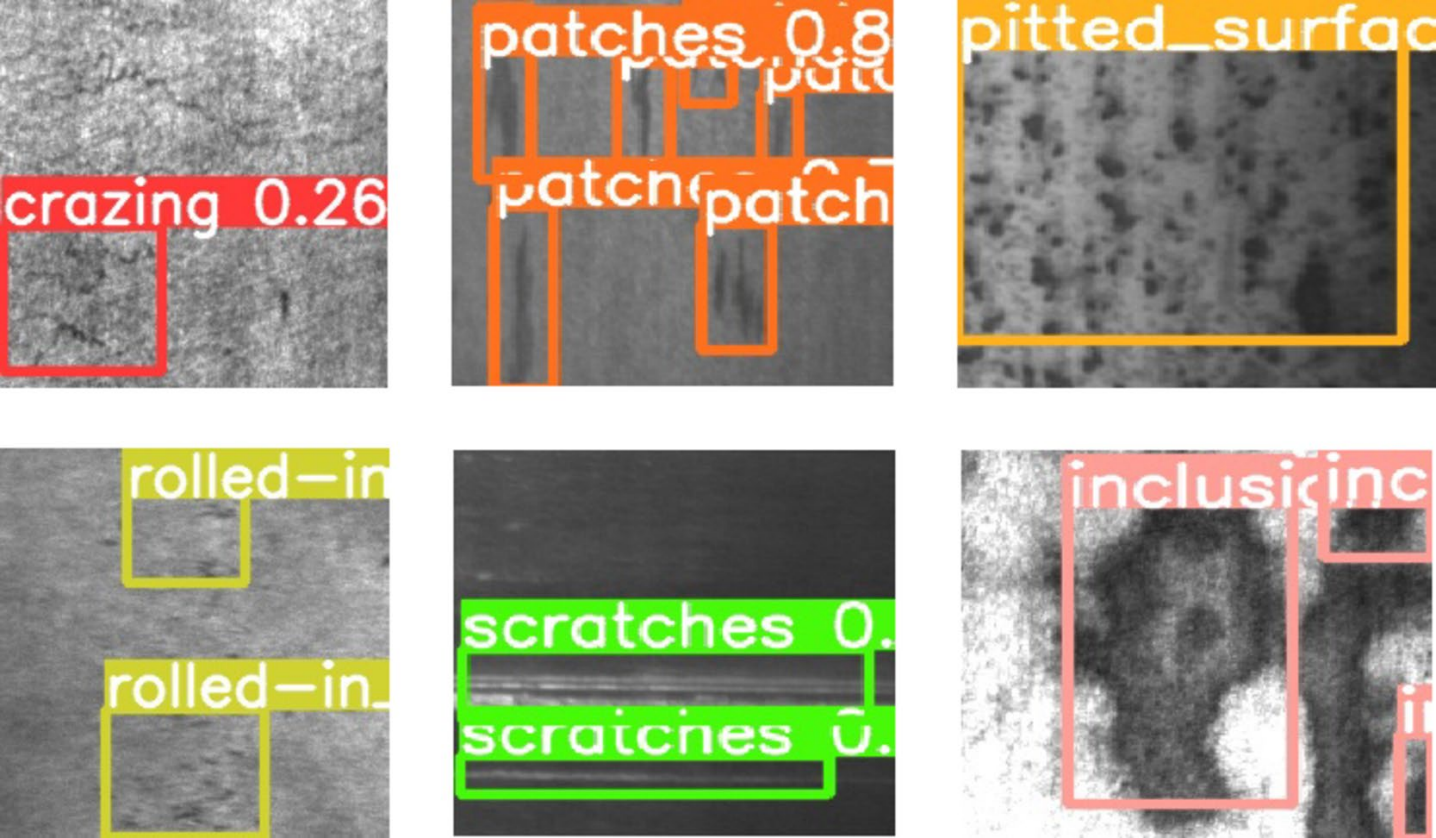
Images of detection result.

The visualization results on the test dataset clearly demonstrate the model’s performance in identifying different types of defects. For instance, elongated defects such as scratches and cracks are detected with high accuracy. However, for smaller defects with unclear boundaries, the model’s performance might be slightly less effective. Additionally, it can be observed that the bounding boxes generated by the model show a certain degree of alignment with the actual defect contours. In most cases, the bounding boxes accurately cover the defect areas, indicating that the model exhibits strong localization capabilities. The detection results are shown in Fig. 11.

**Multiple defects**.

**Fig. 12:**
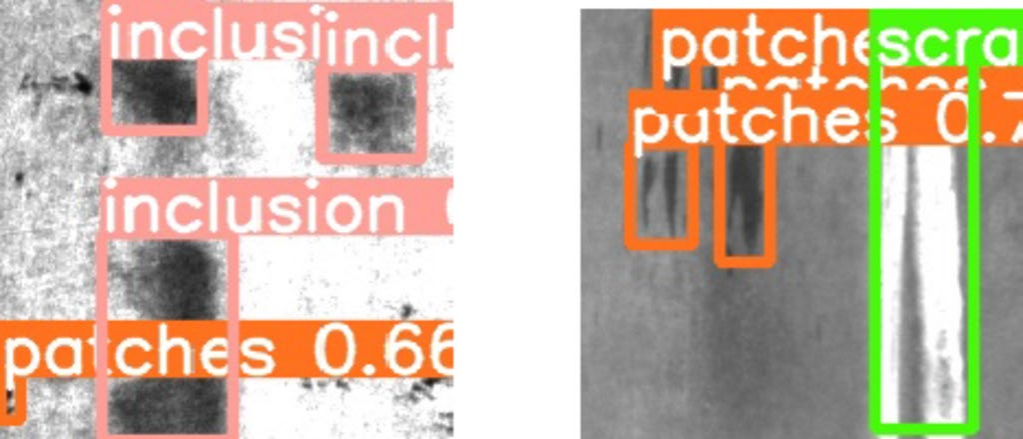
Images of detection result.

In the following examples, it can be observed that multiple types of defects are detected simultaneously. For instance, in a single image, both “patches” and “inclusions” are successfully identified. This demonstrates that the model is capable of handling complex scenarios and effectively distinguishing between defects with different characteristics. The detection results are shown in Fig. 12.

These visualization results indicate that the model exhibits high applicability and robustness in multi-class industrial surface defect detection. However, there is still room for further optimization in addressing complex defect patterns and low-confidence detection results. These analyses provide valuable insights for subsequent model improvements and industrial detection applications.

### Generalization experiments

In practical applications, algorithms often need to handle data of various types, backgrounds, and complexities. Generalization experiments introduce multiple datasets, especially those with different sources and defect types, to comprehensively evaluate the robustness and stability of the algorithm. This ensures that the algorithm can still provide accurate predictions when faced with unseen samples and new problems, which is particularly critical for real-world applications such as industrial defect detection. In such scenarios, models need to address various unforeseen changes and challenges. We first conducted experiments on the NEU-DET dataset, achieving satisfactory results. However, experiments on a single dataset can only demonstrate the model’s performance under specific conditions, making it difficult to reflect its adaptability in diverse environments. Therefore, we further conducted experiments on the GC10-DET dataset to evaluate the algorithm’s performance on industrial defect images with different defect types, shooting conditions, and backgrounds. The GC10-DET dataset is a surface defect dataset collected from real industrial settings. It includes ten types of surface defects: Punching (Pu), Welding Lines (Wl), Crescent Gaps (Cg), Water Spots (Ws), Oil Spots (Os), Silk Spots (Ss), Inclusions (In), Rolled Pits (Rp), Creases (Cr), and Waist Folds (Wf). These defects are all collected from the surfaces of steel plates. The dataset consists of 3570 grayscale images.

The environment and parameter settings were kept consistent throughout the implementation process to ensure the comparability and validity of the experimental results. The results are shown in Table 8.

**Table 8:** Generalization experiments on the proposed improvements.

Method	AP (%)										mAP@50 (%)
	Pu	WI	Cg	Ws	Os	Ss	In	Rp	Cr	Wf	
YOLOv5n	79.5	82.3	85.9	86.3	45.2	49.6	24.3	12.2	60.1	67.2	65.3
YOLOv8s	81.4	86.5	88.4	83.5	42.1	53.5	23.4	12.5	58.1	68.9	65.9
YOLOv9-t	83.4	86.4	91.8	86	45.7	59.4	23.9	11.5	62.2	75.3	66.3
YOLOv10n	80.7	81.9	92.5	82.4	45.9	52.5	23.1	3.8	59.1	68.4	63.2
Proposed algorithm	82.1	86.3	96.1	82.6	48.3	56.9	29	10.9	60.1	72.3	68.4

The experimental results show that, despite the significant differences in defect types, image quality, and shooting angles between the GC10-DET and NEU-DET datasets, the proposed algorithm is still able to effectively identify and classify various types of defects. Specifically, for images with high complexity and diverse defect characteristics, such as crescent gap defects and welding line defects, the algorithm demonstrates stable performance with improvements in both accuracy and recall. This indicates that the proposed algorithm has strong generalization capability when dealing with diverse defects from different data sources and real-world industrial scenarios.

By comparing the experimental results from the two datasets, it is evident that the algorithm exhibits adaptability and robustness across different environments. Although there are some fluctuations in performance across the datasets, the overall trend confirms the model’s strong cross-dataset adaptability. This provides strong support for the further deployment and promotion of the model in real-world applications. In summary, the results of the generalization experiments validate the effectiveness and stability of the proposed algorithm in handling diverse and complex industrial defect detection tasks.

## Conclusion

The healthy development of industrial components industry is directly related to the stability and prosperity of the economy, whether it is military, aerospace, or all aspects of daily life, industrial components play an indispensable role, industrial components surface defect detection in improving product quality and production efficiency are of great significance. Through advanced inspection technology, we can quickly and accurately find and analyze the defects on the surface of components, thus effectively reducing the rate of defective products and improving the stability and reliability of the production line.

Aiming at the problems of low detection accuracy and easy leakage encountered by the current surface defect detection algorithms for industrial components in practical applications, this paper proposes an improved surface defect detection algorithm for industrial parts, and the improved YOLOV5n model has obvious advantages in detection performance compared with the original model and several mainstream target detection algorithms. Compared with the original YOLOV5n model, the mAP@50 is improved by 4.3% with an increase of 3.1 M in the number of model parameters. The model improves the detection accuracy of each defect category to different degrees, among which the defect categories Crazing and Inclusion show the most obvious improvement in detection accuracy. The shortcomings are that the improved scheme has grown a certain number of parameters to the model, and the detection accuracy of the Crazing defect type is still unsatisfactory. In this study, we found that while the model’s accuracy improved, the computation time also increased to some extent. This is mainly due to the increased model complexity and the greater number of iterations required during training. When using more complex convolutional structures and finer training strategies, the model needs more computational resources and time to converge. However, with the support of hardware acceleration (such as GPUs) and other optimization techniques, the increase in computation time can be effectively managed to some extent. Therefore, while the improvement in accuracy may incur a certain computational time cost, this trade-off is acceptable in practice. The application of computer vision technology to solve the surface defect detection problem of industrial parts is of practical significance and provides reference value for defect detection tasks in other industries. The subsequent objectives of my research are: (1) Continue to carry out research to simplify the model structure and reduce the number of model participants. (2) Improve the attention mechanism to speed up the model detection speed and further enhance the model detection accuracy.

## Data Availability

The original datasets used in this study are available in the Northeastern University dataset repository （http://faculty.neu.edu.cn/songkechen/zh_CN/zhym/263269/list/index.htm）. The codes used in this study are available from the corresponding authors.
